# Pan-cancer Analysis of the Disulfidptosis-related Gene NCKAP1 and Its Prognostic Value for Lung Adenocarcinoma

**DOI:** 10.7150/jca.88650

**Published:** 2023-10-09

**Authors:** Ankang Zhu, Yan Zong, Shuai Wei, Yinuo Li, Yan Fan, Shaodong Liu, Xingcai Gao

**Affiliations:** 1 The Fifth Affiliated Hospital of Zhengzhou University, Zhengzhou University, Zhengzhou, China.; 2 Department of Cardiothoracic Surgery, The Fifth Affiliated Hospital of Zhengzhou University, Zhengzhou University, Zhengzhou, China.

**Keywords:** Pan-cancer, immune, Lung adenocarcinoma, prognosis, biomarkers, Transwell.

## Abstract

Background: The nck-associated protein 1 (*NCKAP1*) of the disulfidptosis-related gene is essential in programmed cell death. However, a comprehensive analysis of the biological significance of *NCKAP1* in pan-cancer is lacking.

Methods: Gene expression matrices and clinical expression information of cancers were obtained from The Cancer Genome Atlas (TCGA) and Genotype Tissue Expression (GTEX) databases. A comprehensive analysis of *NCKAP1* expression, biological function, gene mutation, immune cell infiltration, DNA methylation, and drug sensitivity profiles in pan-cancer was performed using the Timer2.0, HPA, GEPIA, STRING, cBioPortal, UALCAN and CellMiner databases. The prognostic value of *NCKAP1* was investigated based on COX regression analysis and the Kaplan-Meier(K-M) curves. A nomogram was established to verify the clinical value of *NCKAP1* for LUAD. The correlation between *NCKAP1* and immune cells and signaling pathways were investigated by single-sample gene set enrichment analysis(ssGSEA). Validation was performed using PCR, Western Blot (WB), and Transwell assays.

Result: Significant differences in expression levels, mutation levels, and methylation levels of *NCKAP1* between tumor and normal samples. *NCKAP1* affects the prognosis of various cancers. *NCKAP1* is strongly associated with microsatellite instability (MSI) and tumor mutational burden (TMB). The Gene Ontology (GO) and the Kyoto Encyclopedia of Genes and Genomes (KEGG) analyses indicate that *NCKAP1* is strongly associated with cell death and tumor immunity. The expression of *NCKAP1* affects the sensitivity to various drugs. Moreover, *NCKAP1* is an independent predictor of prognosis in LUAD patients. The results of ssGSEA showed that elevated *NCKAP1* expression was positively correlated with multiple immune-related signaling pathways. PCR analysis showed that the expression of *NCKAP1* was increased in LUAD cells. Transwell invasion assay showed that overexpression of *NCKAP1* resulted in enhanced invasion of LUAD cells.

Conclusions: We comprehensively analyzed the relationship between *NCKAP1* and pan-cancer and its potential clinical value. *NCKAP1* could be a potential immune marker for various cancers (especially LUAD), providing new insights and insights for cancer therapy.

## Introduction

In most countries, cancer is the leading or second leading cause of death before age 70. Cancer incidence and mortality rates have continued to increase over the past few decades, representing a significant public health challenge worldwide 1, 2. Comprehensive research into common hallmarks of cancer has developed rapidly in recent years, and pan-cancer studies are widely used to explore mechanisms of cancer development and potential biomarkers, which can significantly impact cancer therapy and improve its prognosis 3-6.

Lung cancer ranks second among all cancers and first in mortality, with approximately 2.227 million new lung cancer cases and nearly 1.796 million deaths related to lung cancer in 2020 1. An important reason for this high mortality rate is that more than half of patients have intermediate to advanced lung cancer at the time of initial diagnosis when treatment options are limited and the prognosis is poor 7, 8. Thus, searching for potential immune checkpoints is crucial for treating and diagnosing lung cancer.

In recent years, cell death-related genes have been used to find cures for cancer. For example, the concept of ferroptosis has been widely studied since its introduction in 2012 9. Ferroptosis is an iron-dependent form of regulated cell death progression characterized by excessive lipid peroxidation and subsequent plasma membrane rupture. The association between ferroptosis and cancer has been thought to exist since its inception and has been studied for many years. This observation has been progressively applied to tumor immunotherapy and other areas 9, 10. A recent study has proposed a new mode of cell death, disulfidosis, which refers to cell death in cells with high* SLC7A11* expression due to glucose starvation, resulting in the collapse of disulfide bonds of actin cytoskeletal proteins and actin. This mode of death differs from apoptosis and ferroptosis 11, 12. Preclinical results show that metabolic treatment with glucose transporter inhibitors can cause sulfation and inhibit cancer growth 13. In addition, previous studies have demonstrated that cellular disulfide metabolism is disordered following oxidative stress, which may impact cancer cell survival and proliferation 14, 15. Therefore, it is reasonable to believe disulfidptosis is also closely linked to cancer.

In addition to* SLC7A11*, which plays a vital role in disulfidosis, *NCKAP1* is also a central gene in disulfidosis. The deletion of *NCKAP1* inhibits cell death like *SLC7A11* and attenuates cellular sulfide metabolism 11.

Therefore, we comprehensively analyzed the differential expression of *NCKAP1* in pan-cancer and its impact on the prognosis of pan-cancer. It also explores how *NCKAP1* affects the prognosis of pan-cancer from the gene and immune levels. In addition, we performed drug sensitivity analysis based on *NCKAP1* to guide the clinical application of drugs. We found that LUAD had positive results in several studies of *NCKAP1*, so we focused on analyzing the association between *NCKAP1* and LUAD and demonstrated that *NCKAP1* has the potential to be a therapeutic target for LUAD. Overall, *NCKAP1* is expected to open up new pathways for the treatment of a variety of cancers.

## Materials and Methods

### Data Download

Clinical information and sequencing data for 33 common tumors were obtained from the TCGA database. We also downloaded the TCGA-GTEx data for these tumors to complete the study from UCSC XENA (https://xenabrowser.net/datapages/).

### Expression Analysis of *NCKAP1*

The expression profiles of *NCKAP1* in tumor and normal samples were compared by TIMER2.0 (http://timer.cistrome.org/) 16. Because some tumors did not have normal samples for comparison, we processed and visualized the TCGA-GTEx database sequencing data using the "ggplot2" package within the "R" software package. Protein expression levels of *NCKAP1* in normal tissue and corresponding tumor tissue were obtained from the Human Protein Atlas database (https://www.proteinatlas.org). We also downloaded *NCKAP1* Immunohistochemistry (IHC) images of tumor cells and the corresponding normal cells.

### Prognostic and Survival Analysis

The significance of *NCKAP1* in predicting pan-cancer progression-free interval (PFI) was explored using batch univariate COX regression analysis. Visualize the results with the "forestploter" package in the "R" software. With the help of "Survival" and "SurvMiner" packages in "R" software, we divided the samples into two groups based on the median *NCKAP1* expression: a high-expression group and a low-expression group. Survival curves (based on Overall survival [OS]) for various tumors were plotted using Kaplan-Meier curves.

### Correlation of the infiltration of immune cells with *NCKAP1*

Using the TIMER 2.0 tool, we investigated the correlation between *NCKAP1* expression and immune cell infiltration in different tumor tissues using computational methods such as Timer, EPIC, MCPcounter, CIBERSORT, QUANTISEQ, xCELL, and TIDE. It is worth noting that these algorithms have counted differences in results due to their different working principles. The analysis of the tumor microenvironment receives the influence of multiple factors (different databases, different tumors, differences in detection methods, etc.). These algorithms are applied in different scenarios. TIMER is suitable for estimating the infiltration of multiple immune cell types in tumor tissues and their relationship with tumor development 17. The EPIC algorithm mainly estimates tumor samples' purity and impurity content, providing a basis for subsequent immune cell analysis 18. The MCPcounter algorithm is suitable for estimating the number of different immune and non-immune cell types in tumor tissues and exploring their relationship with tumor progression 19. CIBERSORT is used to analyze the relative abundance of multiple immune cell types in tumor tissues 20. The QUANTISEQ algorithm estimates the abundance of multiple immune cell types in tumor tissues with some flexibility 21. The xCell is suitable for estimating the relative number of multiple immune and non-immune cell types in tumor tissue 22. The TIDE algorithm is mainly used to predict the response to tumor immunotherapy 23.

### Functional Enrichment Analysis

To validate the biological function of *NCKAP1*, we obtained the 100 genes most closely related to *NCKAP1* (in cancer) from the GEPIA database (http://gepia2.cancer-pku.cn). The 100 genes were subsequently studied by GO and KEGG analysis. P-values < 0.05 were considered statistically significant. The "ggplot" package of "R" software visualized the results. We also obtained the top 20 proteins with the highest correlation with *NCKAP1* from the String database (https://cn.string-db.org), and the results were constructed into a network using Cytoscape software. We aim to elucidate the function of *NCKAP1* through these genes and proteins. The functional analysis results of these two gene collections can also be validated against each other to make the findings more accurate.

### The mutation level of the *NCKAP1* and promoter methylation analysis

A comprehensive analysis of *NCKAP1* mutations in 10,967 samples from the TCGA database was performed using the cBioPortal database (https://www.cbioportal.org/). We compared the values of *NCKAP1* methylation in normal and tumor samples using the UALCAN tool (https://ualcan.path.uab.edu/index.html).

### Relationship between *NCKAP1* expression and TMB, MSI

We calculated the TMB of each tumor using the "tmb" function of the "maftools" package and obtained the MSI scores of the tumors from the study of Russell Bonneville et al. 24. Correlations between *NCKAP1* and TMB, MSI were also analyzed separately. The results are listed in Supplementary Table S1. We then visualized the results with the "fmsb" package of "R" software.

### Drug sensitivity analysis of *NCKAP1*

"NCI-60 compound activity data" and "RNA-seq expression profiles" were downloaded from the CellMiner database to analyze the drug sensitivity of *NCKAP1* in pan-cancer. The data were processed using the "impute" and "limma" packages in the "R" package, and drug sensitivity was calculated using Pearson correlation analysis. And |cor|>0.3 and P<0.05 were screening conditions. Finally, the results were visualized by "ggplot2" and "ggpubr".

### Correlation analysis of *NCKAP1* and immune cells and signaling pathways in LUAD

The TCGA-LUAD cohort contained a total of 598 samples. Of these, 539 were tumor samples. 59 were normal samples. To investigate the role of *NCKAP1* in immunity to LUAD. We divided 539 tumor samples into two groups (high and low expression groups) based on the median *NCKAP1* expression values of the 539 samples. Then, we downloaded the immune-related CELLMAKER and signaling pathways from the GSEA website. And ssGSEA analysis was performed on the two groups with the help of the "GSEABase" and "GSVA" packages in the "R" package. We then calculated the correlation between *NCKAP1* expression and several immune checkpoint inhibitors (PDCD1, PDCD2, CD47, CD86, CD276, and CTLA4) in the TCGA-LUAD cohort and visualized the results using the "circlize" package.

### Cell cultuCorrelation analysis of *NCKAP1* with clinical factors

We used univariate and multivariate COX regression analyses to analyze the effects of various clinical factors and *NCKAP1* expression on the prognosis of LUAD patients. The nomogram and calibration curves were then constructed using the "rms" and "survival" packages.

### Cell culture

We purchased Lung epithelial cells (BEAS-2B) and three lung adenocarcinoma cell lines (A549, CALU-3, HCC78), all from Hunan Fenghui Biotechnology Co., cells were cultured on DMEM medium (Gibco, USA) supplemented with 10ml of fetal bovine serum (FBS) (Gibco, USA). The medium was placed in a 37°C, 5% CO2 incubator for stationary culture to reach 80-90% density.

### Reverse Transcription-Polymerase Chain Reaction

RNA was extracted from these four cells using the Redzol reagent (Sebasun, Beijing, China). Synthesis of cDNA using a reverse transcription kit (Toyobo Biotech Co., Ltd., Shanghai, China), and then the mRNA expression level was detected by iQ5 Real-Time PCR (Applied Biosystems, USA). The PCR reaction program was (95°C for 60 s, then 95°C for 15 s, 60°C for 60 s, 40 cycles). All PCR experiments were repeated three times. Finally, all results were normalized by GAPDH. Relative expression levels for these genes were calculated using 2 -ΔΔCt. The primer sequences were: GAPDH, F-5′-TCAGCAATGCCTCCTGCAC-3′, R-5′-TCTGGGTGGCAGTGATGGC-3′. *NCKAP1*, F-5′-TGCTGTAGAAACCCGCAACA-3′, R-5′-TCTGGGTGGCAGTGATGGC-3′.

### Plasmid Transfection Experiment and Western Blot Analysis

The human *NCKAP1* cDNA-containing plasmid was sourced from Shanghai Genechem Co., Ltd. (Shanghai, China). The plasmid was prepared using the pUC ori replicon. Cells were seeded in 6-well plates when the cell density was greater than 80%. Liposome 2000 was used for transfection and subsequently incubated in a 5% CO2 incubator. Transfection efficiency was assessed by Western Blot assay after 48 hours. Cellular proteins were extracted with RIPA lysate. The extracted proteins and markers were separated by electrophoresis with the help of SDS-PAGE gel (Western Protein Marker I: G2086, Servicebio, China). The protein blot was transferred onto a PVDF membrane (Microporous, USA). It was then blocked with 5% skimmed milk. Primary antibodies were incubated at 4 °C for 12 hours (*NCKAP1* antibody: ab126061, GAPDH antibody: ab9485, Abcam, UK). The membranes were washed four times with TBST and incubated with a secondary antibody for 1.5 hours at room temperature (Goat Anti-Rabbit IgG H&L (HRP), Abcam, UK). The cell membranes were placed in Western LightningTM Chemiluminescent Reagent Chromogen (PerkinElmer, USA) for 30 seconds and then immediately placed in an exposure cassette, and finally scanned and imaged with an Epson Perfection V39 scanner, and then analyzed for the expression of each group of protein bands by Image-J software.

### Transwell Invasion experiment

The BD matrix gel was thawed at 4 °C overnight. It was mixed with serum-free medium at 1:8, and 60 μL of diluted matrix gel was aspirated and added to the upper chamber of the Transwell (24-well plate cell chamber of Corning Incorporated, USA). Then, they were incubated in an incubator (37°C, 5% CO2) for 3 hours. The excess liquid in the upper chamber was then aspirated. It was then placed in the incubator for 30 min to hydrate the basement membrane. 500 μL of medium containing 10% fetal bovine serum was added to the lower chamber of the 24-well plate. Then, 200 μL of cell suspension was inoculated into the upper chamber. After 48 h of incubation in the incubator, 4% paraformaldehyde was added to the 24-well plate, and the bottom surface of the chambers was immersed in the solution for 15 min to complete fixation. Excess liquid was subsequently aspirated, and the bottom surface of the chambers was immersed in 0.1% crystal violet for 5 minutes to stain. After cleaning the chamber, the cells were observed under a microscope, counting the top, bottom, right, left and center of the membrane and taking the average.

### Statistical Analysis

The "R" version of the software used in the paper is version 4.3.0. The statistical result P<0.05 was considered statistically significant. (* for* p < 0.05*, ** for *p < 0.01*, *** for* p < 0.001*).

## Result

**Figure 1:**
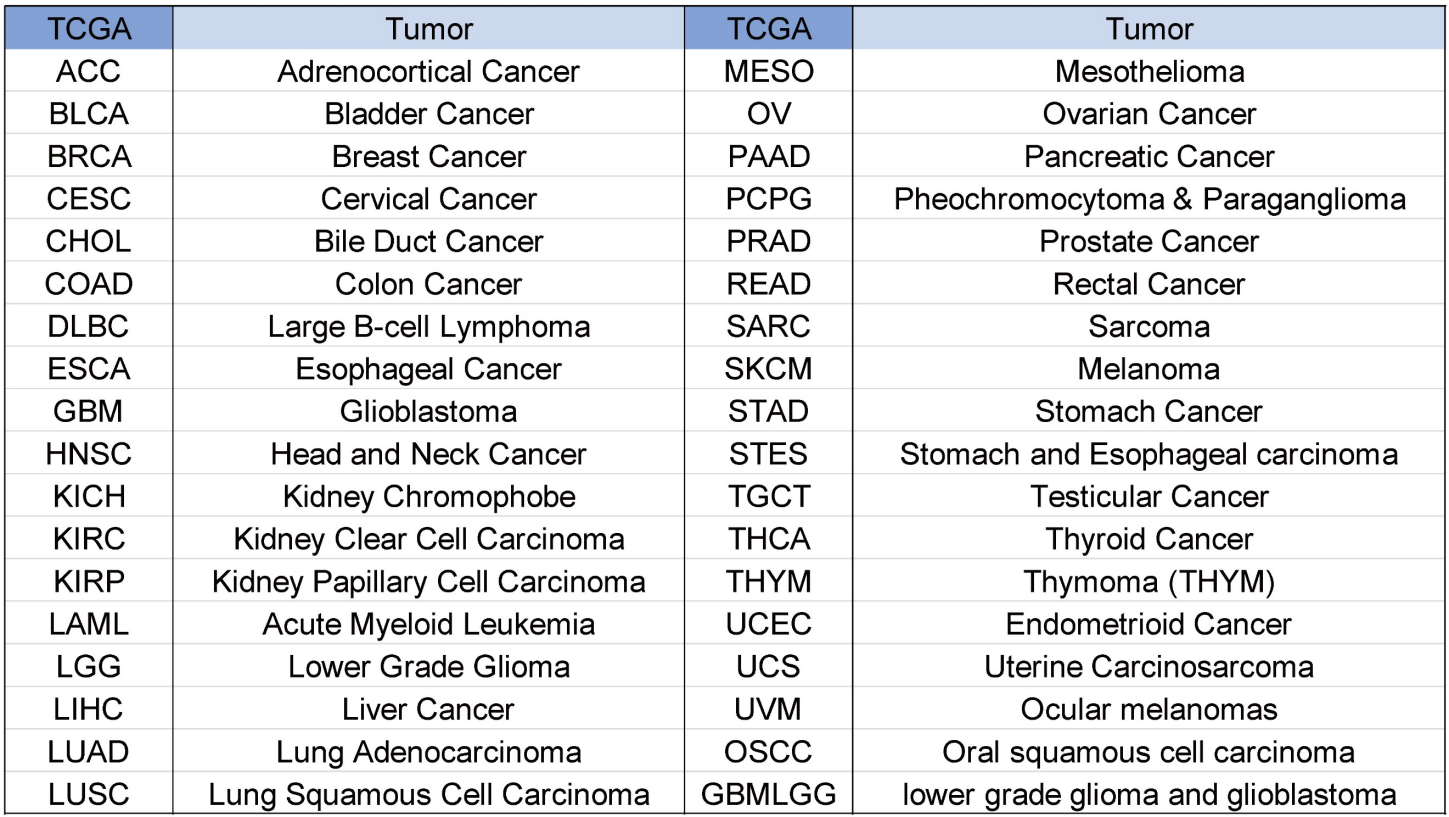
Abbreviations and full names of tumors.

Tumor abbreviations and corresponding full names are shown in Figure 1.

### Expression of *NCKAP1*

**Figure 2:**
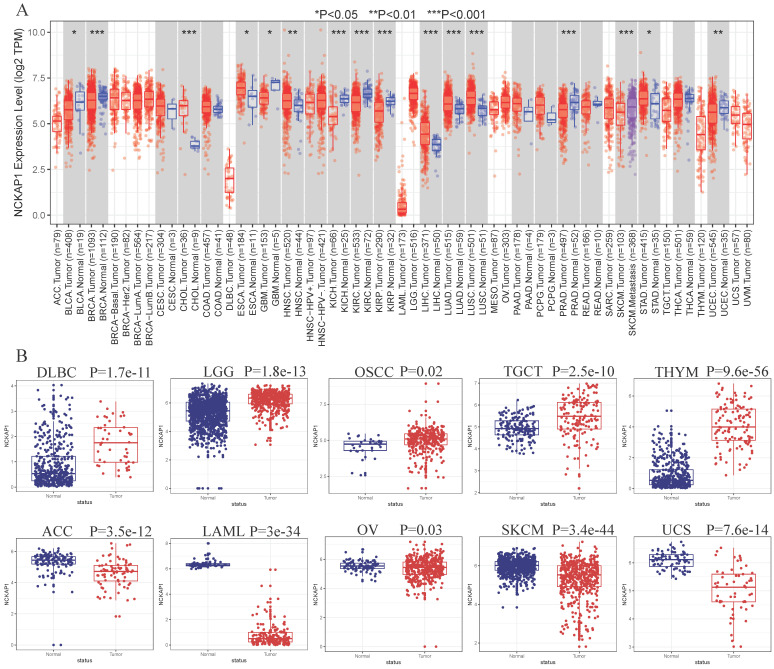
(A) Expression levels of NCKAP1 in pan-cancerous tissues. (B) Differential expression of NCKAP1 in TCGA-GTEX database in multiple cancers (DLBC, LGG, OSCC, TGCTT, THYM, ACC, LAML, OV, SKCM, UCS).

*NCKAP1* expression in tumor and normal samples is shown in Figure 2A-B. Based on the results, we know that *NCKAP1* expression levels were higher in these tumors than in the normal samples, including CHOL, ESCA, HNSC, LIHC, LUAD, LUSC, STAD, DLBC, TGCT, LGG, OSCC, and THYM. In contrast, *NCKAP1* was expressed at lower levels in these tumors than in normal samples such as BLCA, BRCA, GBM, KICH, KIRC, KIRP, PRAD, UCEC, ACC, SKCM, LAML, OV, UCS.

**Figure 3:**
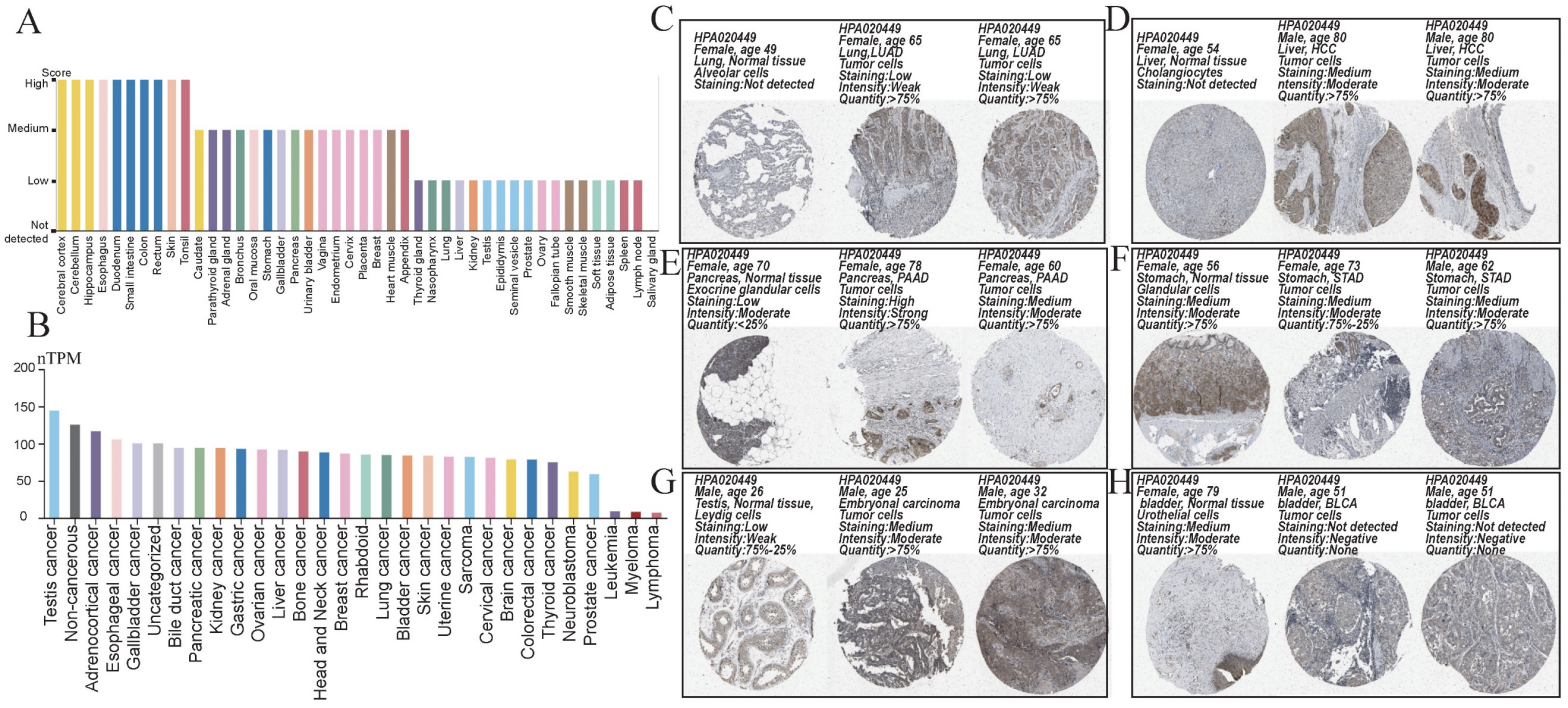
(A) Expression of NCKAP1 in normal tissues. (B) Expression of NCKAP1 in cancer. (C-H) IHC images in multiple cancers and their adjacent tissues.

We obtained a gradient map of *NCKAP1* expression levels in normal tissues and its expression levels in various cancers from the HPA database. *NCKAP1* is highly expressed in the brain, esophagus, and intestine (Figure 3A). Among the cancers, the highest levels of *NCKAP1* were in the testicular, gallbladder, and esophageal cancers (Figure 3B). IHC images were obtained from the HPA database. Lung adenocarcinoma, hepatocellular carcinoma, pancreatic carcinoma, and testicular carcinoma showed higher staining than the corresponding normal samples (Figure 3C, D, E, G). The staining degree of urothelial carcinoma was lower than that of normal tissues (Figure 3H). In contrast, staining in gastric cancer did not differ significantly from normal tissue (Figure 3F). This result is consistent with our analysis above.

### Prognostic analysis

**Figure 4:**
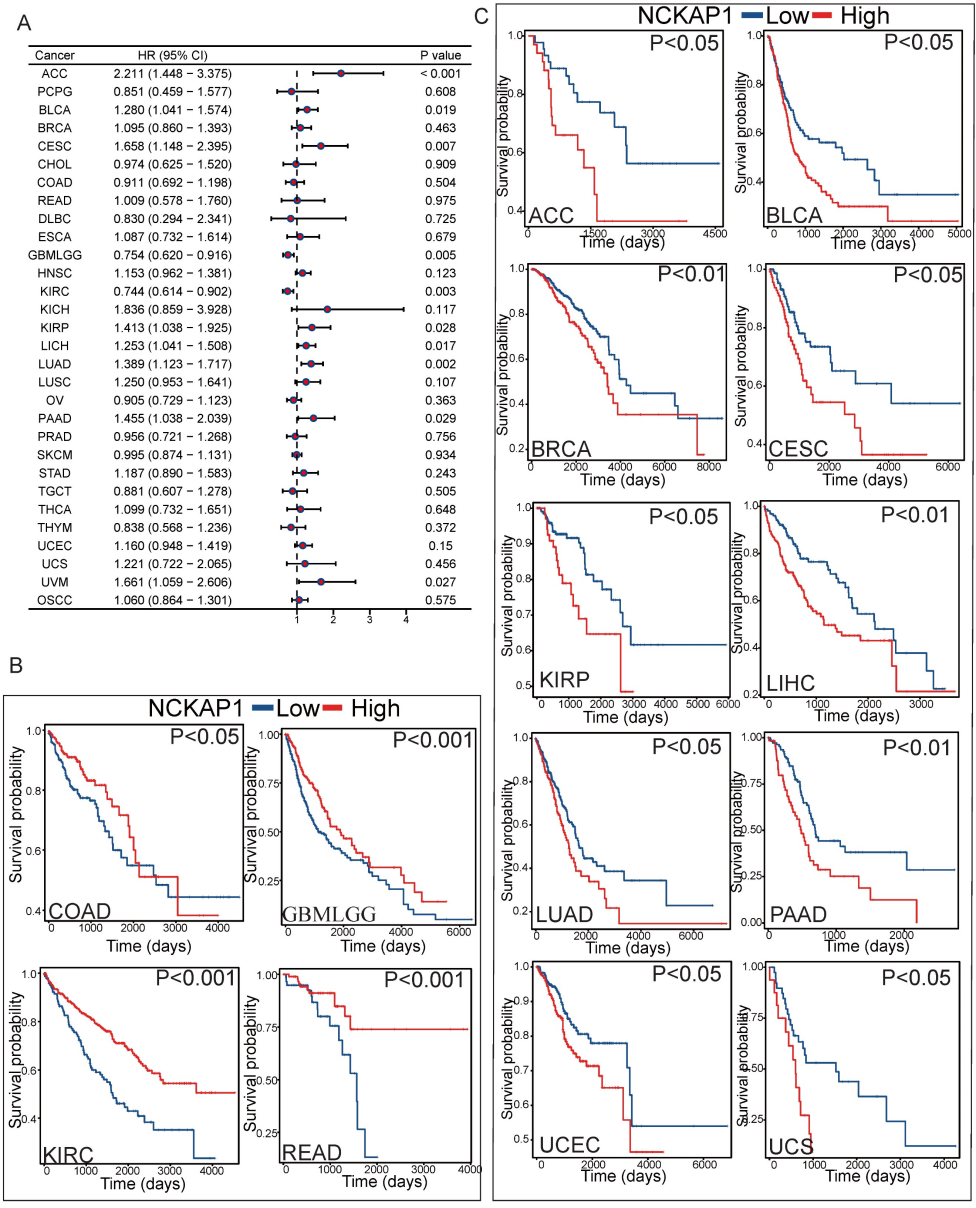
(A) Forest plot showing the results of univariate Cox regression of NCKAP1 on PFI in TCGA pan-cancer. (B-C) Kaplan-Meier curves for NCKAP1 high/low-risk groups in COAD, GBMLGG, KIRC, READ, ACC, BLCA, BRCA, CESC, KIRP, LIHC, LUAD, PAAD, UCEC, UCS.

To further investigate the relationship between *NCKAP1* and tumor prognosis. The relationship between *NCKAP1* expression and PFI in pan-cancer patients was investigated by COX regression analysis. *NCKAP1* was associated with a good prognosis in GBMLGG and KIRC. In contrast, *NCKAP1* was a poor prognostic factor for ACC, BLCA, CESC, KIRP, LICH, LUAD, PAAD, and UVM (Figure 4A). We then plotted the difference in OS between the *NCKAP1* high- and low-expression groups and showed that the low-expression group had a better prognosis in COAD, KIRC, BMLGG, and READ patients (Figure 4B). The high expression of *NCKAP1* resulted in lower OS in ACC, BLCA, CESC, KIRP, LIHC, LUAD, BRCA, PAAD, UCEC, and UCS samples (Figure 4C). These findings suggest that *NCKAP1* does indeed have potential as a prognostic marker. However, in some tumors, there was no clear correlation between the expression level of *NCKAP1* and the survival time (Supplementary Figure S1).

### Correlation between immune cells and *NCKAP1*

**Figure 5:**
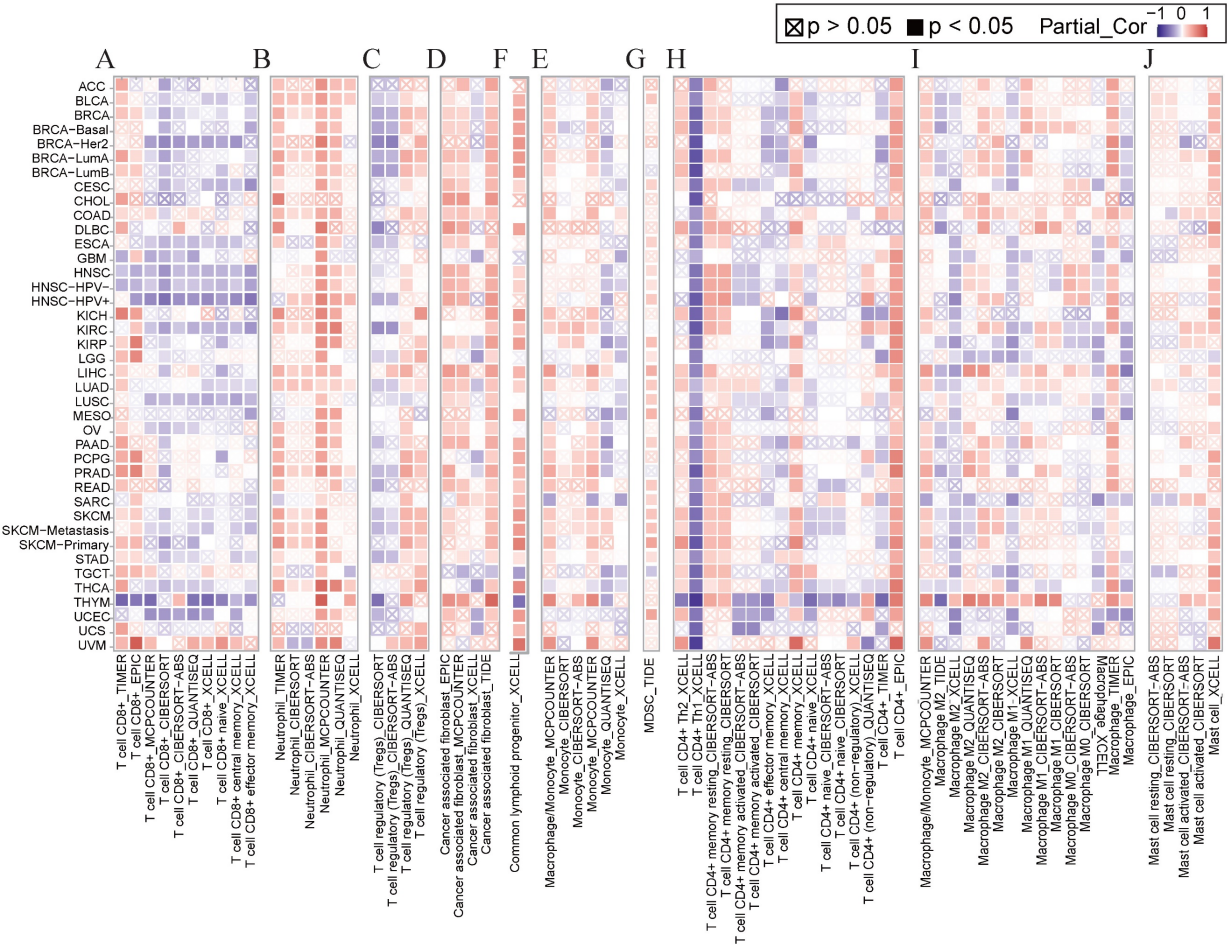
(A-J) Relationship of NCKAP1 with CD8+ T cells, neutrophils, Treg, CAF, CLP, monocytes, MDSC, CD4+ T cells, macrophages, and mast cells in pan-cancer analyzed by multiple algorithms obtained from TIMER2.0.

Several studies have shown that immune cell infiltration into the tumor immune microenvironment is strongly associated with tumorigenesis initiation, progression, and prognosis 25, 26. We applied various algorithms such as EPIC, CiberSort, timer, and XCELL to investigate the relationship between *NCKAP1* and immune cells including CD8+ T cells, neutrophils, T cell regulatory cells (Tregs), tumor-associated fibroblasts (CAF), common lymphoid progenitor cells (CLP), monocytes, myeloid suppressor cells (MDSC), CD4+ T cells, macrophages, and mast cells. In general, *NCKAP1* was positively correlated with the infiltration of a variety of immune cells, such as neutrophils, CAF, CLP, monocytes, MDSC, CD4+ T cells, and giant cells. *NCKAP1* was also found to be negatively correlated with CD8+ T cells (Figure 5). However, the results of different algorithms also varied, and this result needs to be verified in a larger sample.

### GO analysis and the KEGG analysis

**Figure 6:**
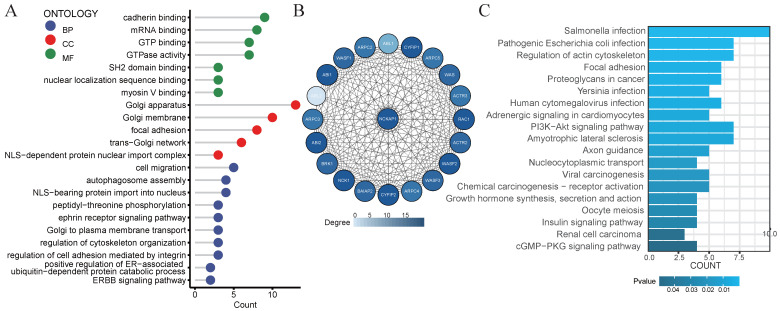
(A) Results of GO analysis of the 100 genes most associated with NCKAP1 expression in pan-cancer. (B) PPI network of the 20 most relevant proteins of NCKAP1. (C) Results of KEGG analysis of the 100 genes most associated with NCKAP1 expression in pan-cancer.

Consider that *NCKAP1* is a crucial gene for disulfidptosis. We decided to explore the biological functions of the *NCKAP1* gene. We obtained the 100 genes with the most significant correlation with NCAKP1 from GEPIA2 (Supplementary Table S2). We performed GO and KEGG analyses on these 100 genes. The results of GO analysis suggested that *NCKAP1* was primarily enriched in calcineurin binding, myosin V binding, regulation of cytoskeletal organization, ERBB signaling pathway, ephrin receptor signaling pathway, and so on (Figure 6A). In contrast, KEGG analysis showed that *NCKAP1* was mainly enriched in the regulation of actin cytoskeleton, focal adhesion, and proteoglycans in cancer, PI3K-Akt signaling pathway, viral carcinogenesis, chemical carcinogenesis - receptor activation, renal cell carcinoma, cGMP-PKG signaling pathway and other aspects (Figure 6C). In light of this result, *NCKAP1* may be known to play an essential role in the cytoskeleton in regulating disulfidptosis. In addition, it is closely linked to various signaling pathways, inflammation, and cancer progression. In addition, we obtained the PPI networks of the top 20 proteins relevant to *NCKAP1* from the STRING database. We visualized the results using Cytoscape software (Figure 6B) and performed GO and KEGG analysis on the 20 genes, and the results matched our functional analysis of *NCKAP1* above (Supplementary Table S3). These observations suggest that the level of *NCKAP1* expression may indeed influence cell death and cancer progression.

### The mutation level of the *NCKAP1*

**Figure 7:**
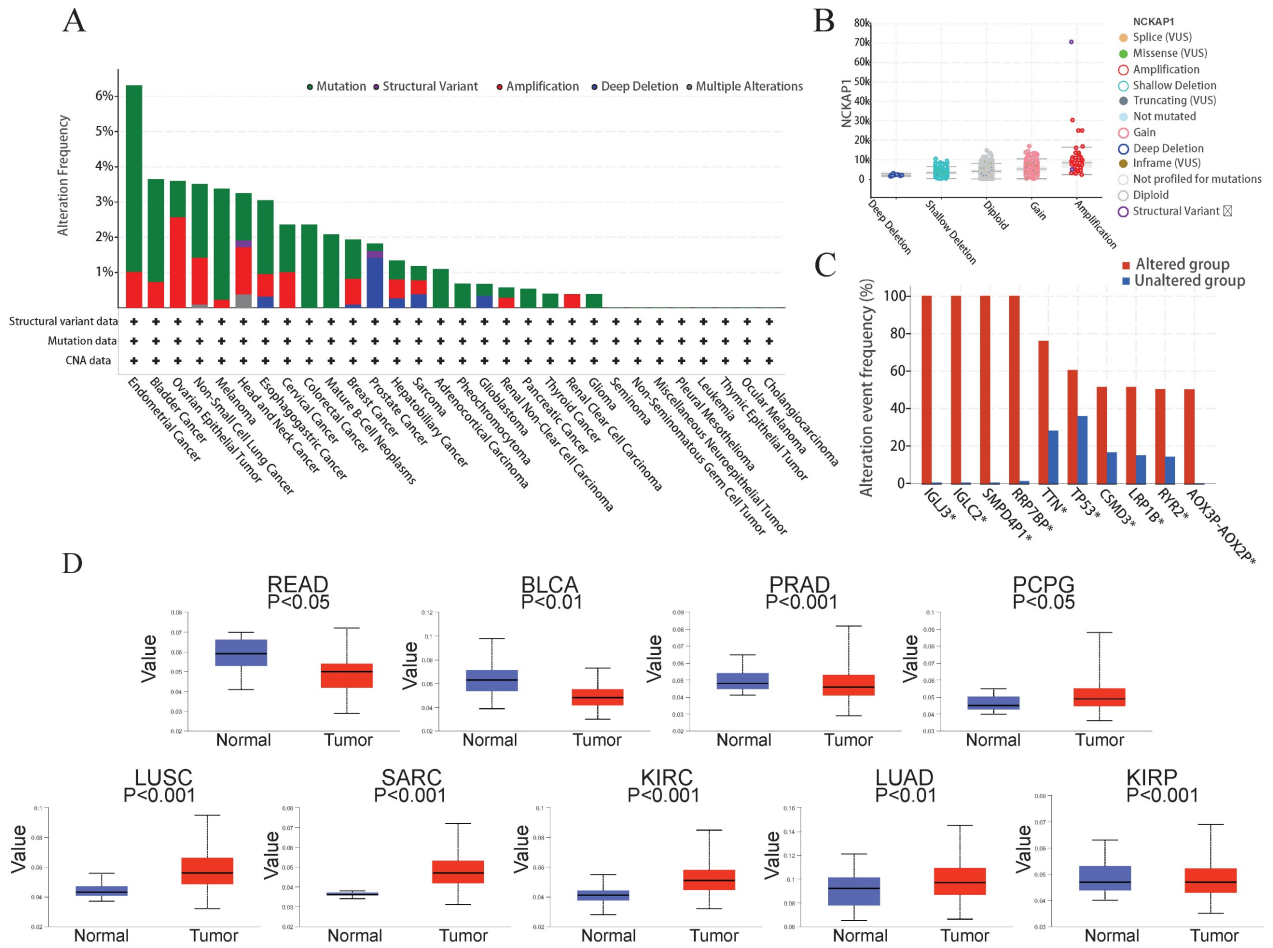
(A) Summary of NCKAP1 mutations in TCGA pan-cancer data. (B) Mutation types of NCKAP1 in pan-cancer. (C) Comparison of the frequency of associated gene alterations in the NCKAP1 altered and unaltered groups. (D) Promoter methylation levels of NCKAP1 in READ, BLCA, PRAD, PCPG, LUSC, SARC, KIRC, LUAD, and KIRP.

Many studies have found that the progression of many cancers is caused by genetic mutations 27. So, we explored the relationship between *NCKAP1* and pan-cancer at the gene level. Using the cBioPortal software, we analyzed mutations in 10 967 samples from the TCGA database and found that mutations in *NCKAP1* are indeed found in various cancers. In endometrial cancer, the mutation rate of *NCKAP1* is even more than 6%. Mutations in this gene were found in over 3% of bladder cancer, ovarian epithelial tumors, non-small-cell lung cancer, melanoma, head and neck cancer, and esophagogastric cancer (Figure 7A). Notably, the primary forms of mutations in *NCKAP1* were amplification and gain (Figure 7B). Alterations in genes such as* IGLJ3, IGLC2, SMPD4P1, RRP7BP, TTN, TP53, CSMD3, LRP1B, RYR2, AOX3P-AOX2P* were more common in the altered group than in the unaltered group (Figure 7C). This situation suggests that *NCKAP1* may act with other genes in tumor progression, which we will analyze specifically in the follow-up.

Several studies have demonstrated that methylation of the DNA promoter can affect transcriptional processes and be involved in tumor development 28. Therefore, we compared the promoter methylation levels of *NCKAP1* in tumor and normal tissues. Consistent with the results shown in Figure 7D, *NCKAP1* promoter methylation levels in READ, BLCA, and PRAD were lower than in the normal samples. Moreover, the levels of *NCKAP1* promoter methylation were higher in the PCPG, SARC, LUSC, KIRC, LUAD, and KIRP samples than in the normal samples, and these differences were statistically significant (*P < 0.05*). These results suggest that the expression of *NCKAP1* in cancer may be receiving the influence of promoter methylation. While comparing some tumors and corresponding normal samples, the methylation levels of *NCKAP1* were also not significantly different (Supplementary Figure S2).

### Correlation of *NCKAP1* with TMB and MSI

**Figure 8:**
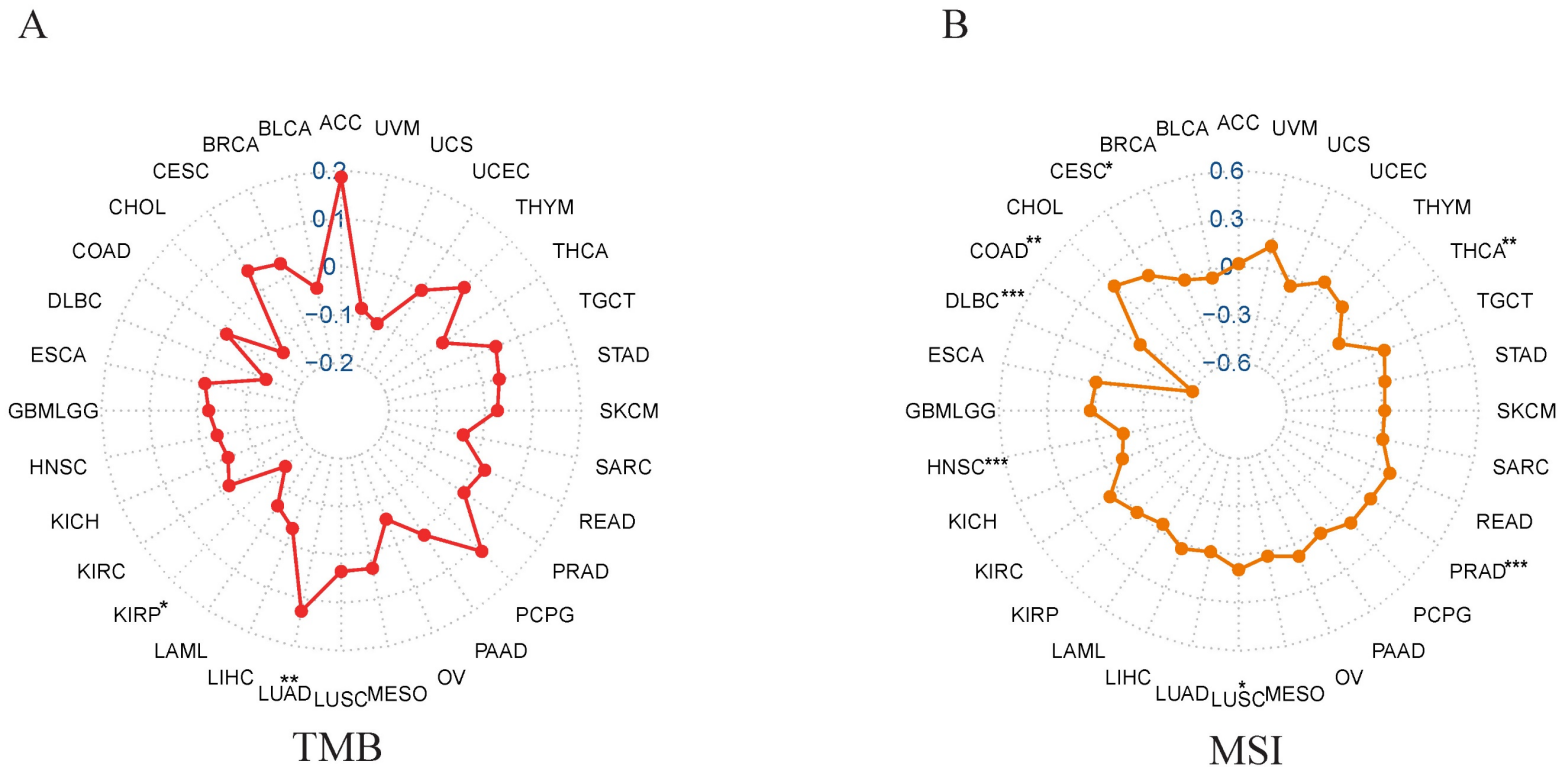
(A) Correlation between NCKAP1 and TMB in pan-cancer. (B) Correlation between NCKAP1 and MSI in pan-cancer.

We analyzed the correlation between *NCKAP1* and MSI/TMB in pan-cancer. From the correlation analysis between *NCKAP1* and TMB, we learned that the expression of *NCKAP1* had a positive correlation with LUAD (*P<0.01*), While *NCKAP1* was found to have a negative correlation with KIRP (*P<0.05*) (Figure 8A). Based on Figure 8B, we observed a positive correlation between *NCKAP1* expression and CESC (*P<0.05*) and LUSC (*P<0.05*). In contrast, *NCKAP1* expression was negatively correlated with the following seven tumors: COAD (*P<0.01*), PRAD (*P<0.001*), HNSC (*P<0.001*), THCA (*P<0.01*), DLBC (*P<0.001*). Specific results are in Supplementary Table S1. These results suggest that *NCKAP1* may influence cancer progression at the mutational level and potentially provide ideas for immunotherapy of tumors (specifically discussed later).

### Drug sensitivity of *NCKAP1*

**Figure 9:**
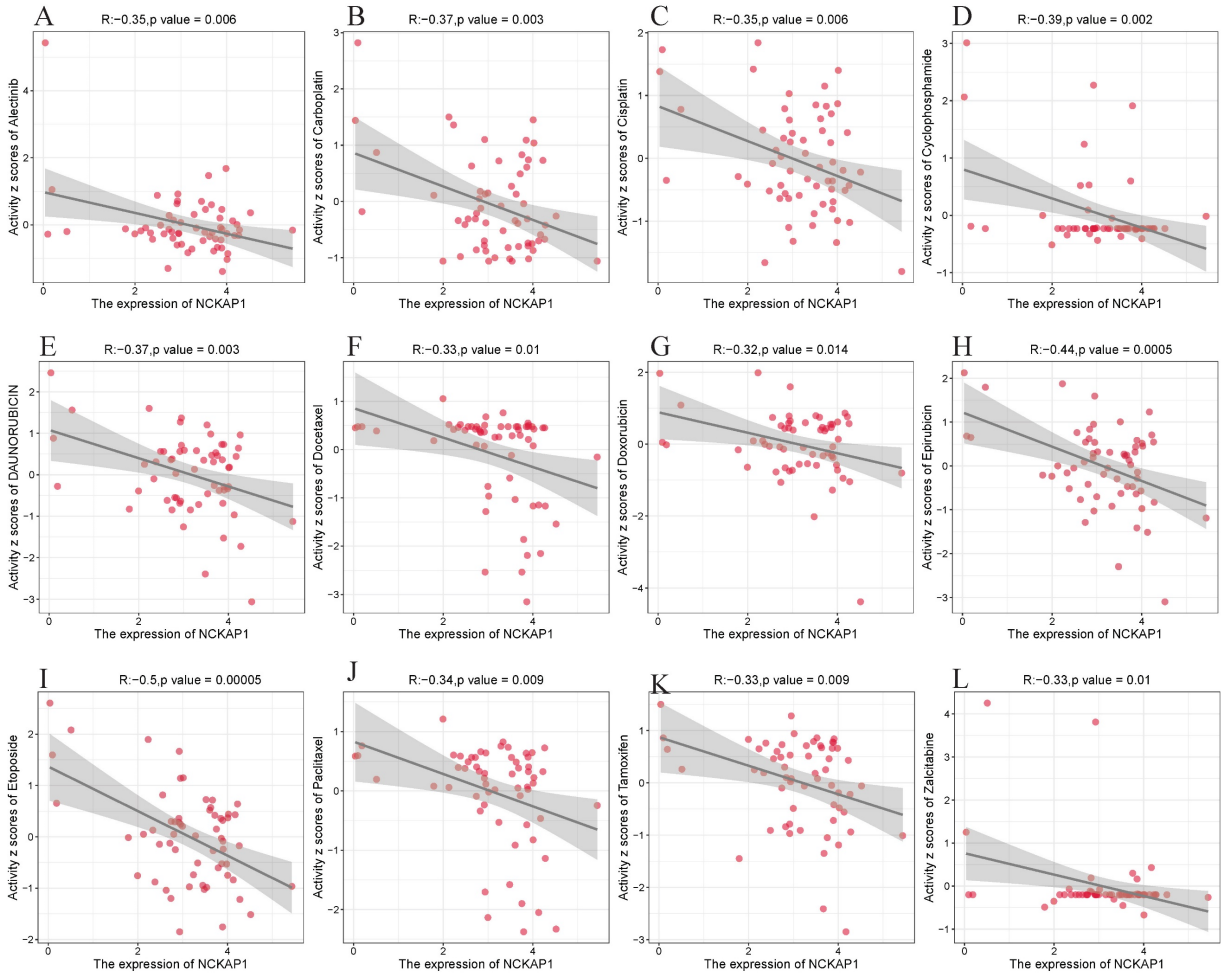
(A-L) NCKAP1 in pan-cancer with drug sensitivity (Alectinib, Carboplatin, Cisplatin, Cyclophosphamide, Daunorubicin, Docetaxel, Doxorubicin, Epirubicin, Etoposide, Paclitaxel, Tamoxifen, and Zalcitabine) analysis.

We explored its clinical value after determining that *NCKAP1* is closely associated with cancer. We analyzed *NCKAP1* with multiple drug sensitivities based on the CellMiner database. After using |cor|>0.3 and P<0.05 as screening criteria, we found 67 drugs with a high degree of association with *NCKAP1*. Of these 67 drugs, 64 were negatively correlated with *NCKAP1*, and three were positively correlated (Irofulven, Kahalide F, Trametinib). Specific results are in the Supplementary Table S4. We chose to visualize the results of several drugs commonly used for chemotherapy in the clinic. *NCKAP1* was negatively correlated with Alectinib, Carboplatin, Cisplatin, Cyclophosphamide, Daunorubicin, Docetaxel, Doxorubicin, Epirubicin, Etoposide, Paclitaxel, Tamoxifen, and Zalcitabine (Figure 9). These findings suggest that *NCKAP1* expression profiling can guide physicians in selecting chemotherapeutics in the clinic.

### Detailed analysis of *NCKAP1* and LUAD

**Figure 10:**
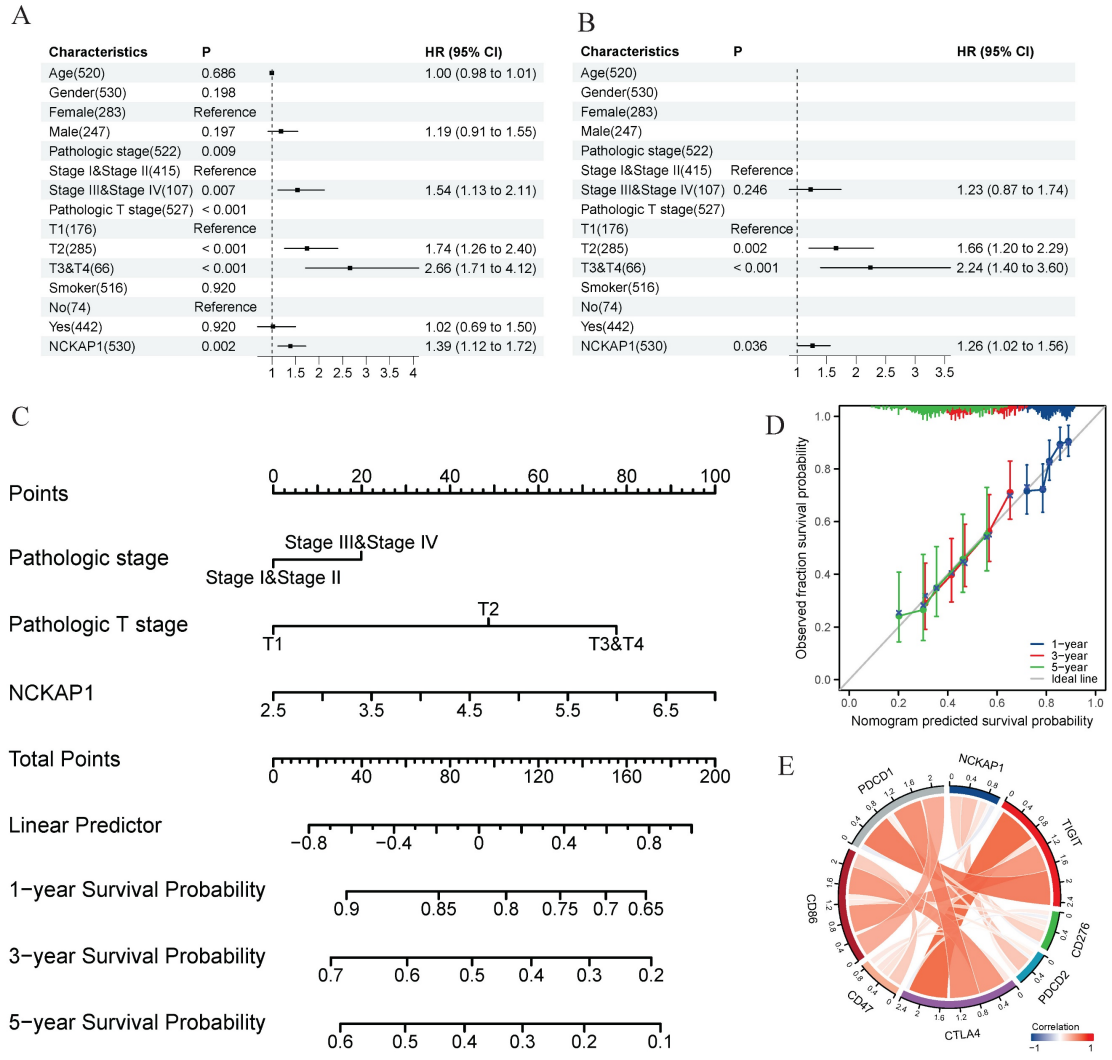
Results of the univariate COX regression analysis (A) and the multivariate COX regression analysis (B) were performed for the TCGA-LUAD cohort. (C) A Nomogram was constructed based on the results of multivariate COX regression analysis. (D)Calibration chart to verify the accuracy of the Nomogram. (E) Correlation between NCKAP1 and suppressive immune checkpoints.

In our pan-cancer study, *NCKAP1* was strongly linked to LUAD. *NCKAP1* is highly expressed in LUAD, leading to a poorer prognosis. To determine whether *NCKAP1* could be a therapeutic target for LUAD. We performed univariate and multivariate COX regression analyses of age, gender, pathological stage, T-stage, smoking or not, and *NCKAP1* expression levels in LUAD patients. The results showed that *NCKAP1* was indeed a factor that independently influenced the prognosis of *NCKAP1* (*P<0.05*) (Figure 10A, B). To further investigate the role of *NCKAP1* in clinical evaluation, we constructed a nomogram based on *NCKAP1*, pathological staging, and T-staging (Figure 10C). We validated the nomogram by calibration curves. The ideal line of the calibration curves graph was our predicted survival of LUAD patients; we found that the 1-, 3-, and 5-year survival curves of the patients almost overlapped with our predicted curves (Figure 10D), which indicated that the performance of the column line graph we built based on *NCKAP1* was good. So, we considered that *NCKAP1* could be a potential therapeutic target for LUAD. We first analyzed the relationship between *NCKAP1* and some important therapeutic targets (PDCD1, PDCD2, CD47, CD86, CD276, and CTLA4) and found that these genes were mainly positively correlated in LUAD (Figure 10E).

### The results of ssGSEA

**Figure 11:**
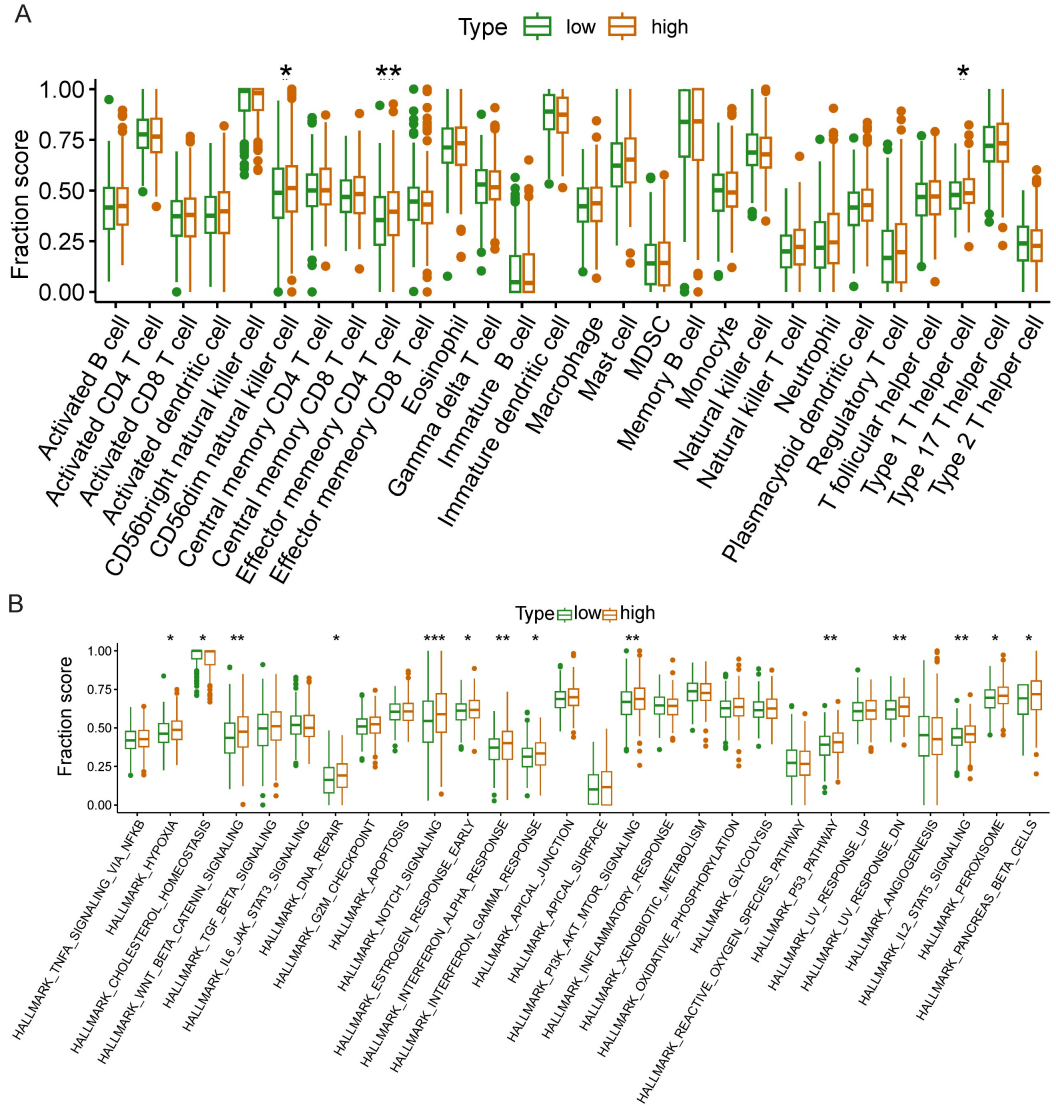
Differences in immune cells(A) and immune-related signaling pathways(B) between high and low expression groups of NCKAP1.

We further investigated whether *NCKAP1* could be used as a therapeutic target of *NCKAP1* by the relationship of *NCKAP1* in immune cells and signaling pathways in LUAD. Based on median *NCKAP1* expression values, we divided 539 TCGA-LUAD patients into high-expression and low-expression groups. We performed ssGSEA analysis to study the correlation between *NCKAP1* and immune cells and signaling pathways. From Figure 11A, we found that the high-expression group had higher enrichment scores in cd56dim natural killer cells, effector memory CD4+ T cells, and type 1 T helper cells. These immune cells play an essential role in the anti-tumor effects of LUAD. This result suggests that high *NCKAP1* expression and tumor immune escape may be related. The results of immune-related signaling pathways showed that the high expression group had higher enrichment scores in these signaling pathways (hallmark_hypoxia, hallmark_wnt_beta_catenin_signaling, hallmark_dna_repair, hallmark_notch_signaling, hallmark_estrogen_signaling, and hallmark_notch_signaling, hallmark_estrogen_response_early, hallmark_interferon_alpha_response, hallmark_interferon_gamma_response, hallmark_pi3k_akt_mtor_signaling, hallmark_p53_pathway, hallmark_uv_response_dn, hallmark_il2_ stat5_signaling, hallmark_peroxisome, hallmark_pancreas_beta_cell) (Figure 11B). These findings suggest that *NCKAP1* can influence LUAD progression via multiple signaling pathways.

### Results of the Experimental Validation

**Figure 12:**
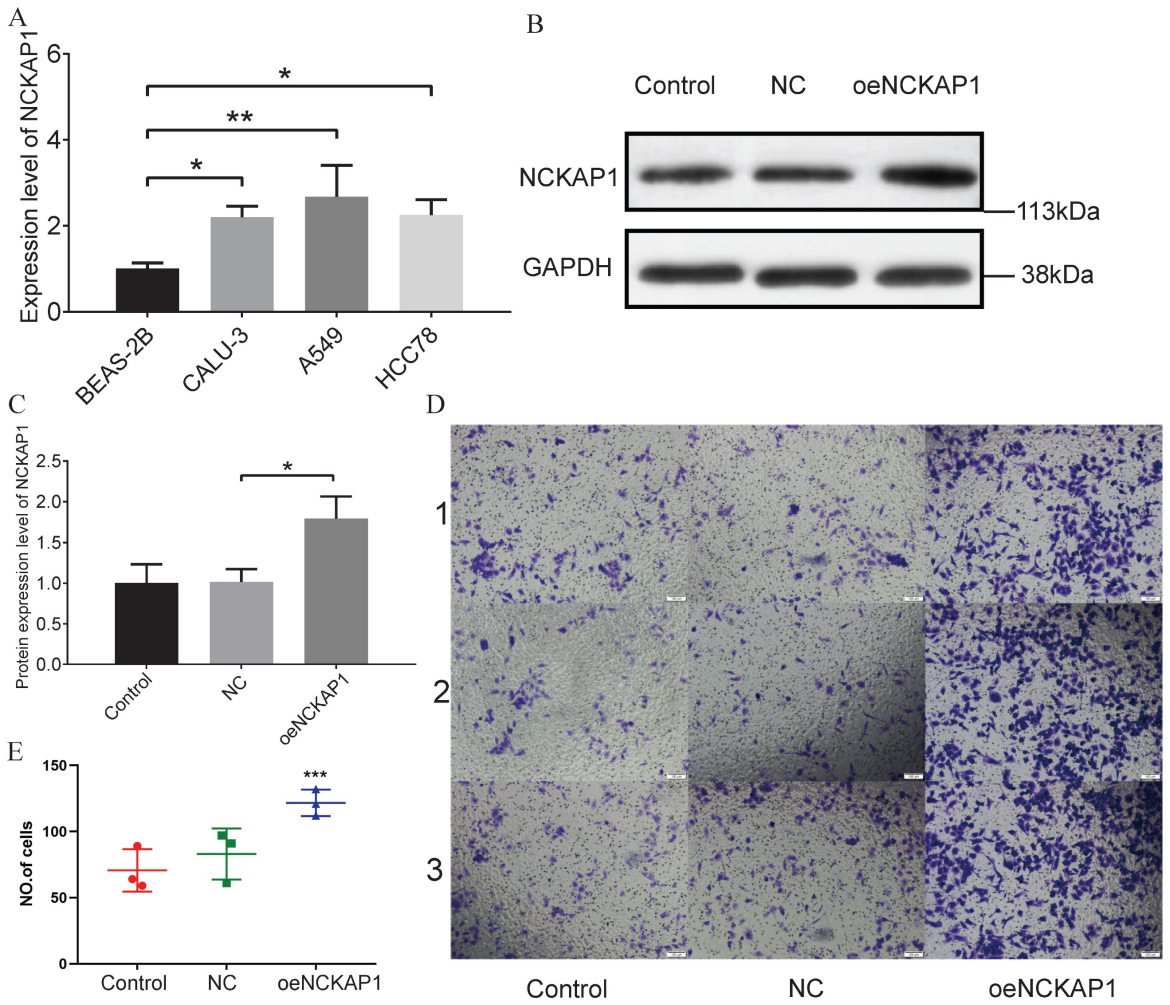
(A) PCR validation of NCKAP1 expression in four cells (BEAS-2B, A549 cells, CALU-3, and HCC78). (B-C) WB results for Control, NC, and oeNCKAP1. Experimental (D) and statistical (E) results of the Transwell invasion test.

Based on the results of PCR experiments, we found that the *NCKAP1* did have a high mRNA expression level in lung adenocarcinoma cells (A549, CALU-3, and HCC78) (Figure 12A). The PCR data are shown in Supplementary Table S5. Then, we randomly divided the A549 cells into three groups. We did not perform any treatment on A549 cells in the first group, named the “Control group.” A blank plasmid was added to the second group of A549 cells, termed the “NC group.” In the third group, we transfected A549 cells with the plasmid that caused *NCKAP1* overexpression and named it “*oeNCKAP1* group”. WB experiments verified our transfection efficiency, and the expression of *NCKAP1* in the *oeNCKAP1* group was higher than in the other two groups (Figure 12B, C). A transwell invasion assay was then carried out on these three groups of cells. The images of the experimental results were also organized and placed in Figure 12D (magnified 100 under the microscope). The statistical results of the experiment are shown in Figure 12E. As can be seen, the invasion ability of A549 cells in the *oeNCKAP1* group was significantly increased. The results of these experiments are consistent with our findings above.

## Discussion

Altered metabolism is an essential hallmark of cancer progression, which often leads to a high dependence of cancer cells on specific nutrients or metabolic pathways that are often breakthroughs in cancer therapy. For example, cuproptosis, a copper-dependent regulation of cell death, is a mode of cell death induced by the toxic stress of tricarboxylic acid (TCA) cycle proteins in mitochondria, which has been progressively used in the treatment and prognosis of many cancers 29. Disulfidptosis is a novel form of cell death in which the signals leading to cell death are recognized by immune cells within the tumor to activate specific immune processes. It alters cellular and humoral immunity and affects the treatment and prognosis of cancer patients. Previous studies have shown that disulfide metabolism in cancer cells affects the biological processes of tumor metastasis and drug resistance 30, 31. And a number of studies have demonstrated the correlation between DRGs and cancers, for example, DRGs can be used in the clinical diagnosis of HCC to predict the prognosis and therapeutic goals 32. DRGs are also closely related to the treatment and prognosis of breast cancer 33.

*NCKAP1* is a critical gene in disulfidptosis and regulates various processes, such as apoptosis, migration, and invasion, and plays a vital role in pathogenesis 34. Therefore, we focused on the expression of the *NCKAP1* gene in various cancers to explore its potential as a potential therapeutic target.

We first investigated the expression of *NCKAP1* in different tumors and normal tissues. The results showed that *NCKAP1* was highly expressed in CHOL, ESCA, HNSC, LIHC, LUAD, LUSC, STAD, SLYC, DLBC, TGCT, LGG, OSCC and THYM. However, the expression of *NCKAP1* was lower in the BLCA, BRCA, GBM, KICH, KIRC, KIRP, PRAD, UCEC, ACC, SKCM, LAML, OV, and UCS. Increased expression of *NCKAP1* in ACC, BLCA, CESC, KIRP, LICH, LUAD, PAAD, and UVM abnormally predicted poor tumor prognosis. These results suggest that *NCKAP1* may be essential in tumorigenesis and prognosis. Therefore, we need further investigate the function of *NCKAP1* in various tumors.

The critical impact of the type and number of immune cells in the tumor immune microenvironment on cancer progression and prognosis is well recognized. In the current study, *NCKAP1* was found to correlate with immune cells, and in particular, the negative correlation with CD8+ T cells sparked our interest. CD8+ T cells play an essential role in the immune response of tumors, and their massive infiltrates are usually considered to portend an improved prognosis 35, 36. Our results suggest that high expression of *NCKAP1* may be associated with the suppression of CD8+ T cells. This point could also explain that aberrant expression of *NCKAP1* leads to poorer prognosis in various cancers. Although our study provides important insights, we would also like to acknowledge the differences in immune infiltration correlations obtained by different algorithms, which may be influenced by the working mechanism of each algorithm and the purpose of the analysis. In addition, immune cell infiltration analysis is also influenced by different databases. However, it is noteworthy that our study observed a consistent trend in some immune cell types (CD8+ T cell, neutrophils, CAF, etc.), and the scope of application of different algorithms varied, which emphasizes the necessity of a multifaceted analysis. In summary, our study provides valuable clues for further investigation of the potential role of *NCKAP1* in tumor immunoregulation. Despite some complexities and discrepancies, our findings remain scientifically essential and provide a solid foundation for more in-depth studies and clinical applications in the future.

We first performed GO and KEGG analysis on *NCKAP1*. The analysis showed that *NCKAP1* is not only a critical gene for disulfidptosis, but it is also closely related to various signaling pathways (cGMP-PKG, ERBB signaling pathway, PI3K-Akt signaling pathway, etc.), inflammation, and cancer progression (viral oncogenesis, chemical oncogene receptor activation, renal cell carcinoma). For example, the PI3K-Akt signaling pathway can promote tumor cell proliferation and metastasis 37. It can also affect the activation and function of T cells, B cells, and macrophages. The abnormally activated PI3K-Akt signaling pathway may inhibit the activity of immune cells, leading to immune escape 38, 39. cGMP-PKG signaling pathway affects tumor immunity by influencing the activation status of immune cells 40. Thus, the multifunctionality of *NCKAP1* in cancer and immunity suggests that it is expected to provide breakthroughs in cancer treatment and immunotherapy development.

We decided to study the relationship between *NCKAP1* and cancer at the genetic level. Gene mutation and DNA methylation have become the focus of attention in recent years through in-depth cancer studies. Genetic mutations are closely linked to cancer's occurrence, development, and metastasis. For example, DNA replication should be considered a hallmark of cancer because it drives cancer progression 41; abnormal upregulation or downregulation of DNA methylation is also believed to contribute to cancer formation and progression or may serve as a marker of cancer development. Hypermethylated DNA is also prevalent in breast cancer 42. Our study found that promoter methylation levels of *NCKAP1* differed in multiple cancers, combined with *NCKAP1* differences in cancers. We suggest that the promoter methylation level influences the expression level of *NCKAP1*. It has been shown that DNA methylation can affect tumor development and biological characteristics by influencing gene transcription 43. The mutation rate of *NCKAP1* in endometrial, bladder, ovarian epithelial, non-small cell lung, melanoma, head and neck, and esophageal cancers was more than 3%. In addition, we found that the mutation rates of *IGLJ3, IGLC2, SMPD4P1, RRP7BP, TTN, TP53, CSMD3, LRP1B, RYR2, and AOX3P-AOX2P* genes were also higher than those of the unaltered group after mutation of the *NCKAP1* gene. This suggests that *NCKAP1* mutations also affect the stability of other genes, thereby influencing tumor progression. Mutations in multiple genes may act synergistically in tumor progression, leading to more complex tumor characteristics. When the mutation rate of multiple genes is increased simultaneously, it indicates that these genes may be involved in common signaling pathways or biological processes. These results will help us gain insight into the effects of *NCKAP1* mutations on tumorigenesis and growth. This finding prompted our decision to explore the association of *NCKAP1* with TMB and MSI in pan-cancer.

TMB is usually defined as the number of genes mutated in the tumor genome. TMB is highly correlated with the efficacy of PD-1/PD-L1 inhibitors, and TMB can be used to predict the prognosis of patients 44. MSI is a feature reflecting microsatellite repeat instability in tumor DNA. As the relationship between TMB, MSI and tumors is complex, we conducted a correlation analysis of *NCKAP1* with TMB and MSI to gain further insight into the immunotherapeutic potential of *NCKAP1* in cancer. Correlation analysis of *NCKAP1* expression with TMB revealed that *NCKAP1* expression positively correlated with LUAD expression (*P<0.01*). This suggests that in LUAD, *NCKAP1* may contribute to poor prognosis by promoting tumor growth and metastasis. So, *NCKAP1* has potential as a therapeutic target for LUAD. However, in KIRP, *NCKAP1* was negatively correlated with TMB (*p<0.05*), but high expression of *NCKAP1* also led to poor prognosis in KIRP. This suggests a possible immune escape mechanism involved by *NCKAP1* in KIRP, and we can target *NCKAP1* to reverse immune tolerance and thus enhance tumor response to immunotherapy. In the correlation analysis between MSI and *NCKAP1*, we found that the expression of *NCKAP1* was positively correlated with the expression of both CESC and LUSC. The expression of *NCKAP1* was negatively correlated with COAD, PRAD, HNSC, THCA, and DLBC. These results suggest that *NCKAP1* may differentially regulate immune features in different tumor types. Further studies will help to reveal the exact role and potential mechanisms of *NCKAP1* in these tumors. These results provide helpful information for individualized immunotherapy and help optimize treatment strategies to improve the efficacy of immunotherapy. Therefore, we will focus on the relationship between *NCKAP1* and the response to specific drug therapy.

Based on the CellMiner database, we sought to determine the drug sensitivity of *NCKAP1* in cancer. Sixty-seven clinically used drugs significantly correlated with *NCKAP1* (Supplementary Table S4). Three drugs showed positive correlations (Irofulven, Kahalide F, Trametinib), which may present a potential therapeutic opportunity. The other 64 drugs showed a significant pairwise negative correlation, including common anti-tumor drugs such as carboplatin, cisplatin, cyclophosphamide, and paclitaxel. Therefore, increased *NCKAP1* expression leads to increased resistance to chemotherapeutic drugs in tumor patients. This study's results will help guide us in using clinical chemotherapeutic agents.

In these study species above, LUAD always had positive results. Therefore, we decided to investigate the relationship between *NCKAP1* and LUAD further. After univariate and multivariate COX regression analyses, we determined that *NCKAP1* was an independent prognostic predictor of LUAD. And based on *NCKAP1*, we established a nomogram to predict the 1-,3-, and 5- survival of LUAD patients. Moreover, *NCKAP1* was positively correlated with common immunotherapy targets. These results suggest the feasibility of *NCKAP1* as a therapeutic target for LUAD. We analyzed the relationship between *NCKAP1* expression and immune cells and immune signaling pathways to explore this potential further. What intrigued our interest was that the *NCKAP1* high-expression group had higher enrichment scores than the low-expression group in multiple signaling pathways. The enrichment of the high expression group in signaling pathways related to immune cell activation (e.g., PI3K-Akt-mTOR, IL-2-STAT5, etc.) suggests that it plays a role in promoting immune cell activation 45-47. Its enrichment in the Notch signaling pathway suggests that *NCKAP1* has an essential role in regulating the immune pathway and may be involved in the differentiation and function of immune cells to influence the host immune response to tumors 48, 49. While hallmark_wnt_beta_catenin_ signaling, hallmark_dna_repair, and hallmark_hypoxia pathways also play essential roles in tumor progression species 50. For example, the Wnt/β-catenin signaling pathway is associated with cell proliferation, differentiation, and stem cell properties, and its aberrant activation has been linked to the development of various cancers 51. *NCKAP1* may also play a role in tumor development and prognosis through these pathways. Enriching these signaling pathways may suggest that *NCKAP1* may be an immunotherapeutic target for LUAD. By interfering with the function of *NCKAP1*, the activation status of immune cells could be adjusted, and the host immune response to tumors could be enhanced.

In conclusion, we investigated the differential expression and prognosis of *NCKAP1* in pan-cancer. By comprehensively analyzing *NCKAP1* in pan-cancer with respect to gene mutation, promoter methylation, and immune microenvironment, we can better understand the function of *NCKAP1* in cancer. We also analyzed *NCKAP1* drug sensitivity in tumors to guide clinical therapy. Finally, we demonstrate that *NCKAP1* plays a role in tumor development and prognosis, with the potential to be a therapeutic target for various cancers. We also found *NCKAP1* as an independent prognostic factor for LUAD and provided a new avenue for immunotherapy in LUAD.

## Supplementary Material

Supplementary tables.

## Abbreviations

TCGA: The Cancer Genome Atlas
TMB: Tumor mutational burden
NCKAP1: Nck-associated protein 1
MSI: Microsatellite instability
KEGG: Kyoto Encyclopedia of Genes and Genomes
GO: Gene Ontology
ssGSEA: Single-sample GSEA
K-M: Kaplan-Meier
WB: Western Blot
OS: Overall survival
IHC: Immunohistochemistry
DRGs: Disulfidptosis-related genes
PFI: Progression-free interval
OS: Overall Survival
Tregs: T cell regulatory cells
CAF: Tumor-associated fibroblasts
CLP: Common lymphoid progenitor cells
MDSC: Myeloid suppressor cells
LUAD: Lung adenocarcinoma
TCA: Tricarboxylic acid
